# A high-throughput method to globally study the organelle morphology in S. cerevisiae

**DOI:** 10.3791/1224

**Published:** 2009-03-02

**Authors:** Shabnam Tavassoli, Jesse Tzu-Cheng Chao, Christopher Loewen

**Affiliations:** Department of Cellular and Physiological Sciences, University of British Columbia

## Abstract

High-throughput methods to examine protein localization or organelle morphology is an effective tool for studying protein interactions and can help achieve an comprehensive understanding of molecular pathways. In Saccharomyces cerevisiae, with the development of the non-essential gene deletion array, we can globally study the morphology of different organelles like the endoplasmic reticulum (ER) and the mitochondria using GFP (or variant)-markers in different gene backgrounds. However, incorporating GFP markers in each single mutant individually is a labor-intensive process. Here, we describe a procedure that is routinely used in our laboratory. By using a robotic system to handle high-density yeast arrays and drug selection techniques, we can significantly shorten the time required and improve reproducibility. In brief, we cross a GFP-tagged mitochondrial marker (Apc1-GFP) to a high-density array of 4,672 nonessential gene deletion mutants by robotic replica pinning.  Through diploid selection, sporulation, germination and dual marker selection, we recover both alleles. As a result, each haploid single mutant contains Apc1-GFP incorporated at its genomic locus. Now, we can study the morphology of mitochondria in all non-essential mutant background. Using this high-throughput approach, we can conveniently study and delineate the pathways and genes involved in the inheritance and the formation of organelles in a genome-wide setting.

**Figure:**
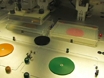


## Protocol

### Materials and methods:

Yeast strains:
  Acp1-GFP (GFP::His): C-terminal GFP-tagging of Acp1 was generated by a PCR-mediated homologous recombination using plasmid pKT128 (contains GFP and *HIS5*). Positive transformants were confirmed by confocal microscopy and colony PCR. The strain background was BY7043 (MAT alpha can1∆::STE2pr-lue2 lyp1∆ his3∆1 leu2∆0 ura3∆0 met15∆0) from Boone lab (Tong and Boone, 2006).
  Deletion Mutant Array (DMA) (xxx::KanR): it is an array of about 5,000 deletion mutants of non-essential genes. All of these nonessential genes were knocked out on G418. The strain background of the array is BY4741 (MATa his3_1 leu2_0 met15_0 ura3_0). Our DMA was originally from Boone lab.
Incorporating Acp1-GFP in the DMA: The following protocol for making a GFP array is adapted from the Synthetic Genetic Analysis (SGA) technology (Tong and Boone, 2006) with modifications. All of the replica pinning steps are done by using a Singer ROTOR high-density arraying robotic system. Alternatively, manual pinning or a liquid-based high-density arraying robot may be used.   Acronyms: YPD, yeast extract-peptone-dextrose; SD, synthetic dropout; LRK, Lue/Arg/Lys dropout media; LHRK, Lue/His/Arg/Lys dropout media  *Preparation*
  Grow a lawn of Acp1-GFP cells by pouring a 5 ml of overnight Acp1-GFP culture onto a YPD plate. Allow the plate to dry sufficiently by a flame or in a biological fume hood.
  Incubate the plate at 30˚C overnight.
  Using a robot, array Acp1-GFP strain into 1536 colonies per plate. At the same time, prepare a fresh copy of the DMA by replica pinning to YPD + G418 plates.
  Incubate the plates at 30˚C overnight. *Mating and diploid selection*
  Mate the Acp1-GFP strain with the DMA by replica pinning and over-laying the colonies on YPD plates.
  Incubate the plates at 30˚C overnight.
  Replica-plate the colonies onto SD – His + G418 plates to select diploid cells (MATa/MATalpha).
  Incubate the plates at 30˚C overnight. *Sporulationg and germination*
  Replica-plate the diploid colonies on *enhanced sporulation media*, which contains reduced levels of carbon and nitrogen sources to induce the formation of meiotic spores.
  Incubate the plates at 25˚C for a minimum of 5 days.
  Germinate the MATa meiotic progenies by replica-plating the colonies onto *LRK media *and incubate the plates at 30˚C for two days.
  This media will induce the germination of haploid cells from spores. Since a *LUE2* opening reading frame  (ORF) is integrated at the MATa-specific STE2 promoter, all the haploids germinated will be MATa. *Selection of both alleles*
  It is now necessary to select both the GFP allele and the single mutant allele. To accomplish this, MATa cells are replica-plated onto *LRK *+* G418 media*, which selects for haploid cells that carry the gene deletion (xxx::kanR).
  Incubate the plates at 30˚C overnight.
  Finally, the MATa meiotic progenies are replica-plated onto *LHRK + G418 media *to select for growth of single mutants (xxx::kanR) also harboring Acp1-GFP (GFP::His). These plates can be stored at 4˚C for up to 3 months.
  The following protocol for making a GFP array is adapted from the Synthetic Genetic Analysis (SGA) technology (Tong and Boone, 2006) with modifications. All of the replica pinning steps are done by using a Singer ROTOR high-density arraying robotic system. Alternatively, manual pinning or a liquid-based high-density arraying robot may be used. Acronyms: YPD, yeast extract-peptone-dextrose; SD, synthetic dropout; LRK, Lue/Arg/Lys dropout media; LHRK, Lue/His/Arg/Lys dropout media  *Preparation*
Microscopy:
  Pick the desired mutant(s) expressing Acp1-GFP directly from plate.
  Grow cells at 30 °C in YPD or SD – His media to early log phase.
  Visualize by confocal microscopy using the GFP filterset. An example of expected result is show in Figure 1.

#### SGA media:

The growth media (YPD) and selecting media (SD) used in this protocol is routinely used in yeast molecular biology. Please refer to *Methods in Yeast *(Amberg et al., 2005) for detailed descriptions.

#### LRK, LHRK  media (for 400 ml total)

**Table:** 

Add in order	Amount
Yeast nitrogenous base with ammonium sulfate	2.7 g
Amino acid mix	0.8 g
Agar	8 g
dH_2_O	360 mL
Autoclave
20 % Dextrose	40 ml
Cool to 65˚C
100 mg/ml canavanine	0.2 ml
100 mg/ml thialysine	0.2 ml
Mix, pour 50 ml per plate

#### LRK, LHRK + G418 media (for 400 ml total)

**Table:** 

Add in order	Amount
Yeast nitrogenous base without ammonium sulfate	0.7 g
Amino acid mix	0.8 g
Monosodium Glutamate	0.4
Agar	8 g
dH_2_O	360 mL
Autoclave
20 % Dextrose	40 ml
Cool to 65˚C
100 mg/ml canavanine	0.2 ml
100 mg/ml thialysine	0.2 ml
200 mg/ml G418	0.4 ml
Mix, pour 50 ml per plate

#### Enriched sporulation media (for 400 ml total)

**Table:** 

Add in order	Amount
Potassium acetate	4 g
Yeast extract	0.4 g
Dextrose	0.2 g
Sporulation amino acid mix	0.04 g
Agar	8 g
dH_2_O	360 mL
Autoclave
Cool to 65˚C
200 mg/ml G418	0.4 ml
Mix, pour 50 ml per plate

## Discussion

This method can help efficiently incorporate a mitochondrial marker, Acp1-GFP into various mutant backgrounds. It relies on the use of a robotic system, and can easily adopted for use with any robotic system. This procedure can be also used for incorporating other types of markers. For example, to visualize ER, we routinely use the marker Erg11-GFP. In our representative images, a mutant and a wild type with the Acp1-GFP maker were visualiz ed by confocal microscopy to study the mitochondria morphology. The mutant showed disrupted mitochondrial phenotype, and is therefore a good candidate for further investigation.
